# The Retinol Circulating Complex Releases Hormonal Ligands During Acute Stress Disorders

**DOI:** 10.3389/fendo.2018.00487

**Published:** 2018-09-04

**Authors:** Yves Ingenbleek

**Affiliations:** Laboratory of Nutrition, Faculty of Pharmacy, University Louis Pasteur, Strasbourg, France

**Keywords:** lean body mass, transthyretin, retinol-binding protein, thyroid function, cytokines, retinoids, stress disorders, immune responses

## Abstract

Intensive care workers actively participate in very hot debates aiming at defining the true metabolic, hormonal and nutritional requirements of critically ill patients, the contributory roles played by thyroid and retinoid ligands being largely underestimated. The present article makes up for redressing the balance on behalf of these last hormonal compounds. The retinol circulating complex is transported in the bloodstream in the form of a trimolecular edifice made up of transthyretin (TTR), retinol-binding protein (RBP) and its retinol ligand. TTR reflects the size of the lean body mass (LBM) and is one of the 3 carrier-proteins of thyroid hormones whereas RBP is the sole conveyor of retinol in human plasma. In acute inflammatory disorders, both TTR and RBP analytes experience abrupt cytokine-induced suppressed hepatic synthesis whose amplitude is dependent on the duration and severity of the inflammatory burden. The steep drop in TTR and RBP plasma values releases thyroxine and retinol ligands in their physiologically active forms, creating free pools estimated to be 10-20 times larger than those described in healthy subjects. The peak endocrine influence is reached on day 4 and the freed ligands undergo instant cellular overconsumption and urinary leakage of unmetabolized fractions. As a result of these transient hyperthyroid and hyperretinoid states, helpful stimulatory and**/**or inhibitory processes are set in motion, operating as second frontlines fine-tuning the impulses primarily initiated by cytokines. The data explain why preexisting protein malnutrition, as assessed by subnormal LBM and TTR values, impairs the development of appropriate recovery processes in critically ill patients. These findings have survival implications, emphasizing the need for more adapted therapeutic strategies in intensive care units.

## Introduction

TTR is a highly conserved protein mainly found in the choroid plexus [CP] and in the liver in all classes of vertebrates. TTR is synthesized and secreted by the CP in reptiles, birds and mammals whose TTR gene is believed to have been turned on in the CP at the stage of the stem reptiles (1). Synthesis of TTR in the liver occurs much later during organogenesis in all vertebrate species and continues during adulthood in birds, some marsupials and eutherian mammals (2).

TTR was identified in human CSF in 1942 (3) and in human serum in 1956 (4). In humans, TTR is one of the 3 specific binding proteins [BPs] ensuring the transport of thyroid hormones [TH] (5) in addition to albumin and thyroxine-binding globulin [TBG]. TTR is a molecule comprising 4 identical subunits each displaying a sequence of 127 amino acids [AAs] that coalesce noncovalently in parenchymal cells to yield a nascent tetramer with a 55 kDa molecular mass [MM] (6). The 4 subunits form an open channel harboring 2 binding sites for TH (7). One of the TTR monomers transports a small companion protein displaying an inner cleft in which a single binding site for one molecule of all-*trans*-retinol is located, hence its name, retinol-binding protein (RBP, 21 kDa) (8). Each RBP molecule may bind a single retinol ligand to form an holo-RBP complex that may undergo further aggregation with TTR within the liver endoplasmic reticulum, yielding a trimolecular retinol-circulating complex [RCC] edifice totaling 76 kDa in MM (9). In vitamin A-replete subjects, the 3 components remain attached within close stoichiometry 1: 1: 1 in spite of their different biological half-lives [T_1/2_ of 2 days for TTR (10) vs. half a day for RBP (11)], showing that TTR safeguards holo-RBP from premature urinary output and serves as a limiting factor for the delivery of retinoid compounds to target tissues (12). In accordance with specific tissue requirements, holo-RBP molecule releases its retinol ligand to peripheral tissues, a process reducing its affinity for TTR (13). The further loss of a terminal arginine AA residue [174 Da as MM] yields an apo-RBP derivative (14) that demonstrates a significantly lowered T_1/2_ of approximately 3.5 h (15). Apo-RBP rapidly crosses the glomerular barrier prior to its disintegration within the renal tubules. Recycling of the resulting AAs is an efficient process, as shown by the barely detectable concentrations of apo-RBP molecules measured in the urinary output of healthy subjects. The main characteristics of TTR, RBP and apo-RBP molecules are shown in Table 1.

**Table 1:** Main physico-chemical and metabolic characteristics of TTR, RBP, and Apo-RBP (Healthy reference man weighing 70 Kg).

**Parameter**	**TTR**	**RBP**	**Apo-RBP**
Molecular mass (Daltons)	54,980 (6)	21,200 (8)	21,026 (14)
Conformation	Tetrameric	Monomeric	Monomeric
AA sequence	4 × 127 (6)	182 (8)	181 (14)
Carbohydrate load	Unglycosylated	Unglycosylated	Unglycosylated
Normal plasma values	270–330 mg/L	50–65 mg /L	—
Biological half-life (T $\frac{1}{2}$)	2 days (10)	14 h (11)	3.5 h (15)
Plasma value of bound ligand (B)	80 μg TT_4_/L	500 μg retinol/L	—
Plasma value of free ligand (F)	2 ng FT_4_/L	1 μg FR/L	—
F/B ratio	1: 4,000	1: 500	—
Distribution space of free ligands	12 L (16)	18 L (17)	—

*References of original publications are cited in square bracket*.

**Figure 1:**
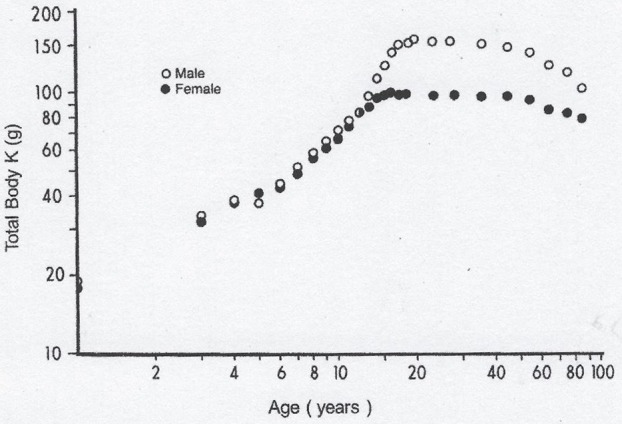
Evolutionary patterns of LBM values throughout the human lifespan. Compilation of 7 different clinical investigations performed in healthy subjects from birth to very old age and showing body accretion TBK values determined by the measuremement of the naturally occuring radioisotope ^40^ K using dual-energy X-ray absorptiometry (DXA). The results are plotted against age on double-logarithmic coordinates. Ninety-five percent of TBK is sequestered within metabolically active tissues and narrowly correlated with total body N (TBN), making this last parameter a valuable tool to appraise LBM values in health and disease (18). Figure shows that normal TBK concentrations are approximately 140–160 g in adult men and 90–110 g in adult women.

**Figure 2:**
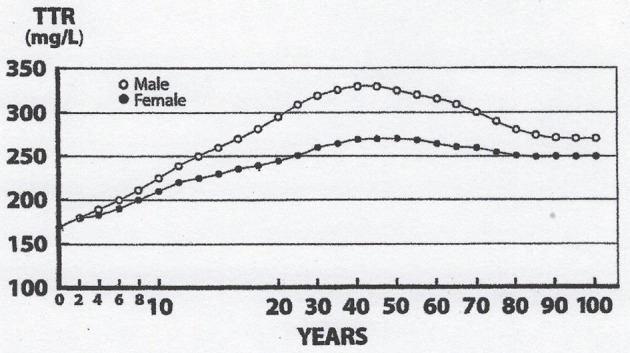
Evolutionary patterns of TTR concentrations throughout the human lifespan. TTR concentration are measured in the blood samples on 67,720 healthy US citizens using immunoturbidimetric analysis (19). TTR and LBM values manifest closely superimposable trajectories: Lowest values measured at birth, linear progression without sexual difference until the onset of puberty, occurrence of sexual dimorphism with more pronounced rise in adult males because of a large musculature, followed by plateau levels until the age of 60y, and finally gradual downsizing toward sacropenia in both sexes with a steeper slope observed in elderly males. Both TTR and LBM curves show comparable abrupt S-shape elevations from the onset of adolescence until the beginning of adulthood, which are here partially obliterated due to changes in the graduation of the abscissa scales.

Lean body mass [LBM] results from a composite agglomeration of fat-free tissues [(18), Figure 1] displaying with TTR closely superimposable developmental patterns from birth to old age [(19), Figure 2], making this BP a reliable surrogate tool to assess fluctuations of LBM size in clinical investigations (20). At birth, plasma TTR concentrations increase linearly without sexual difference during infant growth (21). Human puberty is characterized by major hormonal and metabolic alterations leading to significant redistribution of body tissues (22), most notably the muscle mass which is the main component of LBM by weight. As a result of deeper androgenic impregnation, a significantly higher S-shaped elevation of LBM has been recorded in male adolescents compared with the blunt curve documented in teen-aged girls (23). A comparable gender dimorphism is observed during the onset of adolescence for TTR values, which display developmental patterns (19) tightly matching those outlined by LBM (18). In healthy adults, the sex-related differences in plasma TTR and RBP concentrations are maintained in the form of plateau levels during full sexual maturity (19). Normal TTR plasma values are stabilized around 300–330 mg/L in males and approximately 250–270 mg/L in females whereas RBP plasma values manifest a similar sexual difference at approximately 63 and 52 mg/L, respectively. Beginning in the sixties, the muscle mass undergoes downsizing, with a steeper slope in elderly men, accounting for the concomitant decline in LBM (18, 23) and TTR-RBP values (19). The LBM includes the bulk of total body protein stores and may schematically be subdivided into a *visceral* compartment comprising metabolically active organs characterized by rapid turnover rates (liver, small intestine mucosa, thymoleukocytic tissues) and a *structural* compartment distinguished by organs manifesting slower metabolic activity (skeletal muscle mass, skin, cartilages, connecting tissues, appendages) (24). Keeping in mind these body composition characteristics, we here report on the roles played by TTR and RBP as reservoirs of ligands released and converted into bioactive derivatives that have survival impact during the acute phase of any inflammatory condition.

### TTR and thyroid hormones in inflammatory disorders

In a reference man weighing 70 kg and living under steady state conditions, protein homeostasis is the net result of protein synthesis equaling protein breakdown. The renewal rate of LBM is evaluated at 3% day^−1^, meaning that approximately 300 g of protein are lost and regenerated every day (25). In the case of inflammatory disorders, activated leukocytes release a shower of cytokines that act as autocrine, paracrine and endocrine molecules (26). Cytokines govern the overproduction of acute-phase reactants [APRs], which contribute in several ways to defense and repair mechanisms using specific kinetic and functional properties (27). Interleukin-_6_ [IL-_6_], interleukin-_1_ [IL-_1_] and tumor-necrosis factor_α_ [TNF_α_] are regarded as key mediators of acute and chronic inflammatory processes, and are not expressed under normal circumstances explaining why APR concentrations are maintained at baseline levels (27). IL-_6_-nuclear factor (NF) possesses a high degree of homology with C/EBP-NF_1_, a nuclear transcription factor that function via a retinoic acid response element [RARE] present in its promoter region. C/EBP-NF_1_ competes for the same DNA response element of the IL-_6_ gene, causing a dramatic elevation in APR values under stress conditions (28), together with a reciprocal suppressed TTR synthesis as demonstrated in animal (29) and clinical (30) experiments. The abrupt decline in plasma TTR values has major functional implications, the importance of which has been until now significantly underrated in stress disorders. The liver serves as a large storage site for thyroxine, and is capable of harboring as much as 30% of the total extrathyroidal T_4_ body pool (31). The liver also secretes 3 specific BPs that ensure the normal transportation of total thyroxine (TT_4_) into the intravascular space [5 L]. Despite minor disagreements, it is generally thought that TBG carries approximately 70% of TT_4_ whereas TTR and albumin equally share the carriage of the remaining 30%. According to the free hormone hypothesis and to the law of mass action (32), any endocrine ligand is metabolically inactive as long as it remains attached to the binding sites of specific carrier protein(s). This applies to TT_4_ since only minute fractions of its ligand are released in the free form [FT_4_] and may exert hormonal activity (33). The normal TT_4_ plasma value is 80 μg/L, whereas that of FT_4_ is 20 ng/L, implying a free [F] to bound [B] ratio of 1: 4,000. In the case of acute surgical or inflammatory stress of medium severity, TTR plasma values are usually decreased by 40% within 4 or 5 days, indicating that the 12 μg of thyroxine transported by TTR should release about 5 μg of FT_4_. The first demonstration that the fall in the TTR plasma values is responsible for increased free thyroxinemia in postoperative states was described as early as in 1964 (34), but this original observation was not acknowledged by the scientific community for a long time, mainly because FT_4_ undergoes instant cellular overconsumption allowing to meet increased metabolic requirements of inflamed tissues.

Freed ligands are now recognized to uniformly diffuse within their normal distribution space of 12 L (16) to reach an estimated FT_4_ concentration of 400 ng/L throughout the critical stress period, meaning about 20-fold the normal FT_4_ value. This hormonal alteration thus creates a transient hyperthyroid state that is upheld by the measurement of significantly higher plasma FT_4_ values in infected (35) and surgical (36, 37) patients. At least 3 other stress conditions may contribute to filling the thyroxine distribution space with an additional supply of FT_4_ ligands: (1) bacterial infections cause a 4-fold accelerated peripheral turnover of both thyroid hormones (38), (2) anesthesia and surgery mobilize FT_4_ (39) from the large hepatic T4 extrathyroidal reservoir (31), and (3) suppressed synthesis of TBG contributes to the enlargement of the pool of bioavailable FT_4_ fractions (40). In surgical patients, declining TTR and rising FT4 concentrations demonstrate mirror image of each other (39) with FT4 peak values reached on day 4 after initial impact (36). As a result of FT_4_ flooding, molecules exceeding body tissue needs remain unmetabolized and undergo renal leakage (41), consistent with the view that increased thyroxinuria faithfully reflects the overall thyroid status (42). This hyperthyroid context has significant functional impact as disclosed by experiments using two-dimensional radioautography that indicate that approximatively 8% of the liver mRNA products are modified under TH influence (43). The biological activity of TH depends on serum hormonal levels, on the roles exerted by at least 3 major cytosolic transporters, by intracellular deiodinases and by a large family of nuclear receptors that modulate target gene expression (44). As a result, TH regulates the basal metabolic rate as well as the activity of enzymes and of most protein and carbohydrate components that participate in the immune adaptive responses to stress disorders (45). Most of these activities are mediated through the activation of transcription factors that have stimulatory influences (46, 47) whereas some sustain inhibitory effects as shown by thyroid hormone-induced abrogation of TBG synthesis (40). Several relevant biomarkers produced along this transitory hyperthyroid environment are shown in Table 2 (45, 48–58).

**Table 2:** Stimulatory effects of thyroid hormones in inflammatory disorders.

**Target mechanisms/tissues**	**Induced effects**	**References**
Cellular immunity	Control of innate and adaptive immune responses	(45)
T-cell immunity	Growth and distribution of thymocytes in lymphoid organs	(48)
Liver injury	Enhanced mitosis of liver cells following hepatectomy	(49)
Liver sepsis	Increased survival of hepatocytes under stress conditions	(50)
Repair mechanisms	Boosting effects on hepatic synthesis and release of IGF-1	(51)
Erythrocytes	Stimulatory effects on ferritin synthesis and hematopoiesis	(52)
Cartilage	Maturation of chondrocytes promoting matrix calcification	(53)
Joints	Differentiation and growth of cartilaginous tissues	(54)
Epidermal growth factor	Binding and phosphorylation of liver EGF receptors	(55)
Nerve growth factor	Stimulation of NGF mRNA activity	(56)
Cerebral tissues	Maturation and differentiation of brain cell lines	(57)
Brain cell survival	Upsurge of brain-derived neurotrophic factor (BDNF)	(58)

### RBP and retinoid derivatives in inflammatory disorders

Unlike TTR, holo-RBP is the sole conveyor of retinol in human plasma whose normal concentration in an adult man (60 mg/L) ensures the carriage of approximately 500 μg retinol /L whose free fraction (FR) is measured at 1 μg/L, hence a normal F:B ratio of 1: 500. In the case of an acute stress of medium severity that decreases the holo-RBP concentration by 40%, an estimated amount of 200 μg retinol is released as FR, diffusing uniformly in a larger distribution space of 18 L (17) than that of FT_4_ and yielding an augmented concentration estimated at 10–12 μg/L during the critical illness period, meaning about 10-times the normal free value. To our knowledge, no study has described the sequential upsurge of plasma FR in stress disorders up to now, but clear indirect evidence exists indicating that this adaptive elevation rests on firm grounds since unexpected retinoluria is recovered after surgical stress (41), febrile rotavirus diarrhea (59), shigellosis (60), and enterotoxic Escherichia colitis (61). Healthy subjects do not excrete detectable amounts of retinol, RBP or apo-RBP in the urine, suggesting that their increased urinary values measured following acute stress (41) result from expanded extracellular free pools whose unmetabolized fractions undergo overflow into the kidney. This retinoluria must be regarded as a reflection of the duration and severity of injury that occur transiently and passively without leading to kidney dysfunction. The delivery of retinol targets a variety of cell-surface receptors manifesting uneven distribution in body tissues, with a high concentration found in the epithelial cells of the CP and organs belonging to the visceral compartment (liver, small intestine, bone marrow) (62). The discovery of a specific cell surface receptor stimulated by retinoic acid 6 [STRA6] that recognizes holo-RBP and mediates the uptake of its retinol ligand (63) has prompted novel investigations involving the JAK/STAT signaling cascade and RBP-dependent insulin resistance pathway (63, 64). Interestingly, plasma TTR may block the association of holo-RBP with its STRA6 receptor and thereby exert protective effects on RBP-induced insulin resistance (63, 64). These data suggest that subnormal TTR plasma values, as seen in protein-depleted states (20), could refrain from interfering with the STRA6-mediated retinol uptake process and ensuing events, thereby reinforcing insulin refractoriness. The biological significance of these metabolic peculiarities in morbid conditions such as diabetes, obesity, cancer (64, 65) and protein malnutrition (20) needs to be elucidated. In addition, nonspecific intracellular transfer of FR has also been described (66). After crossing the cell membrane, retinol undergoes several conversion steps leading to specific derivatives. Whereas 13-*cis*-retinal is the required compound that is involved in the visual cycle (67), all-*trans*-retinoic acid is regarded to be the principal inducer of intracellular effects of vitamin A in mammalian tissues (68). The first study describing nuclear receptors for retinoid compounds (69) has launched an identification of the genomic signaling effects of vitamin A and a better understanding of the pleiotropic activities exerted at cytosolic level (68, 69). Retinoids modulate the differentiation of epithelial T cells (70), the regulation and maturation of B cells (71), notably via the stimulation of cytokines (72), T-killer cells (73) and the balance between Th-1 and Th-2 types of T-cells (74). Likewise, TH and retinoid compounds released in their free form throughout an acute stress reaction manifest a large preponderance of stimulatory activities on effector tissues and may even work in concert with TH to potentiate immune effects by mediating nuclear mechanisms (75, 76). Retinoid compounds may also trigger inhibitory effects by fine-tuning the adequacy of acute phase responses or by depressing the activity of pro-inflammatory mediators such as IL-_3_ (76), IL-_12_, and TNF_α_ (77). Some of the main cellular effects triggered during hyperretinoid states are summarized in Table 3 (70–81), focusing more specific attention on the mechanisms implicated in repairing body tissues injured during stress disorders (75, 76, 80, 81).

**Table 3:** Stimulatory effects of retinoid compounds in inflammatory disorders.

**Target mechanisms/tissues**	**Induced effects**	**References**
T - cell immunity	Maintenance of T-cells and attenuation of inflammation	(70)
B-cell proliferation	Regulation and differentiation by retinoic acid	(71)
Cytokine induction	Stimulatory overproduction of interleukins - 1 and - 3	(72)
T-cell immunity	Cellular induction via T-killer cell induction	(73)
RA-receptors	Dualistic influences on Th1 and Th2 development	(74)
Keratin genes	Maturation of filament proteins forming keratin	(75)
Red blood cells	Combined effects of TH and RA on erythropoiesis	(76)
RA effects on leukocytes	Antagonistic effects of Il-_10_, Il-_12_ and TNFα	(77)
Activated B – cells	Promotion of CD_4_(+) T cell differentiation	(78)
CD_8_ T-cell activation	Modulation of T-cells differentiation by RAs	(79)
Epidermal growth factor	Control of EGF binding and mitogenic activities	(80)
Skin fibroblasts	Proliferation of extracellular matrix of dermal fibroblasts	(81)

The above-described clinical example applies to patients afflicted with stress disorders of medium severity. In the case of more harmful injuries such as polytrauma, multiple organ failure or burns (82, 83), the cytokine-induced fall in the TTR-RBP plasma concentrations entails longer slopes in connection with the magnitude and duration of LBM breakdown. The nadir point may be regarded as reflecting the residual LBM reserves below which the adequacy of further inflammatory responses may be compromised. The downward TTR-RBP drop is an *obligatory* process refractory to any nutritional manipulation. Extensive thermal injury represents one of the most inflicting hypercatabolic states identified by TTR-RBP values falling to about 20–30% of starting levels with a nadir point reached on days 6–8 after the initial impact (83), implying higher and persisting impregnation of target tissues with freed ligands resulting in super-activated APR responses.

### Re-orchestration of energy and endocrine requirements

The above-mentioned data emphasize that thyroid and retinoid pathways maintain complex interactions and multifaceted cross-talks in stress disorders, operating in combination or antagonistically. Here, it is necessary to take into account the roles played by these freed ligands in the context of other metabolic activities controlled by the so-called counter-regulatory hormones. These adaptive responses to stress lead to the dichotomous partitioning of metabolic requirements between healthy and inflamed tissues that are described elsewhere in minute detail (84). The energy requirements of healthy tissues are moving toward lipid dependency (RQ~0.7), thereby sparing glucose and AA residues (25). Acute stress disorders are followed by a post-impact period of time characterized by the onset of a euthyroid sick syndrome (85) generated by Il-_1_, Il-_6_ and TNFα (86–88) which work in concert to negatively affect all functional steps of the hypothalamo-pituitary-thyroid axis, notably via inhibition of 5′- monodeiodinase activity (5′-DA), thereby hindering intrathyroidal conversion of FT_4_ to its more potent derivative, FT_3._ As a result, the drop in plasma FT_3_ values is accompanied by the maintenance of energy expenditure and protein anabolic processes at the minimally required levels (89).

Inflamed tissues manifest opposite endocrine and metabolic patterns. Ambient hyperglycemia favors the simple diffusion of glucose into the core of injured and poorly irrigated tissues, allowing for the support of the local anabolic drive via anaerobic glycolysis and low oxygen consumption (RQ~1) (25). The liver escapes the overall context of receptor refractoriness and governs the uptake of AAs redirected into the preferential production of defense and repair processes under the control of glucagon and TNF_α_. Glucocorticoids govern the major acute phase response in liver cells (27, 28), as documented for the synthesis of both C-reactive protein (CRP) (90) and α_1_-acid glycoprotein (α_1_-AGP) (91), the latter also being under TH-dependency (40). The dichotomous partitioning of energy and substrate requirements is further illustrated by the dualistic behavior of freed IGF1 fractions, the mitogenic processes of which are downregulated in the whole body economy (92) but intensified in injured territories (93, 94). Likewise, the aforementioned cytokines operate together to impair intrathyroidal expression and activity of 5′-DA (86–88) and allow the body to meet peripheral tissue requirements in a specific manner that results in a beneficial outcome (95). Taken together, the data show that the TH ligands released in the free form develop peak endocrine and metabolic effects at the site of inflammation, using specific cytokine mechanisms among which are abrogation of liver synthesis (40), proteolytic breakdown of carrier-proteins (93, 94) and enzymatic regulation of intrathyroidal T4 to T3 converting processes (86–88). TTR and RBP clearly play central roles in the dichotomous partitioning and re-orchestration of metabolic priorities associated with the development of adaptive responses to stress disorders.

## Conclusions

TTR is a plasma protein endowed with a number of functional properties and is now widely used both in developing areas for the nutritional assessment and follow-up of underprivileged populations and in developed countries to identify those hospitalized patients at risk of protein-deficiency. The close links recently established between TTR and LBM developmental patterns in health and disease (20) have renewed interest in the biomarker now regarded as a unique and robust indicator of protein nutritional status. Protein malnutrition is a common finding in hospitalized patients typically characterized by defective cytokine production (96). The amount of ligands released in their free form is determined by the difference between pre- and post-stress TTR-RBP values. The liver production of these last carrier-proteins (12, 97) undergoes rapid down-regulation in protein-depleted states, explaining why significantly blunted APR responses are recorded when malnutrition coexists with superimposed inflammation (98–100). TTR plasma concentrations situated below 200 mg/L constitute a threshold below which serious clinical complications are predicted to occur (101) whereas a boundary of 100 mg/L carries ominous prognostic significance (102). We sustain the view that FT_4_ and FR fractions released during the acute period of stress should help to throw a fresh look at the overall hormonal climate associated with the rehabilitation of injured patients. Without contesting the validity of the massive increase in FT_4_ and FR values recorded during the 1970s in critically ill patients, it is nevertheless necessary to keep in mind that comparable data have not been described recently. At the present time, most investigators participate in very hot debates in intensive care units, and aim to stay close to the true metabolic and nutritional requirements of critically ill patients. The scope encompasses energy and protein needs, the roles played by specific AA residues, fatty acid fractions, vitamins and oligo-nutrients. Hormonal compounds clearly play central roles in this highly controversial issue (103). Authors renowned for having published authoritative monographs on GH (104) and insulin (105) have paved the way for novel and more relevant approaches, as shown by the well-documented detrimental effects generated by the exogenous administration of GH (106) contrasting with the beneficial impact of added insulin which allows normalization of harmful hyperglycemia and attenuation of the levels of cytokines, APRs and most pro-inflammatory biomarkers (107). The under-diagnosed stimulatory or inhibitory activities exerted by TH and retinoids in stress disorders combining malnutrition and inflammation influences should prompt workers to enlarge the spectrum of more appropriate preventive and therapeutic strategies. This recommendation is founded in the light of recent data showing that increased FT4 values may have deleterious effects on the cardiovascular system (108).

## Author contributions

The author confirms being the sole contributor of this work and approved it for publication.

### Conflict of interest statement

The author declares that the research was conducted in the absence of any commercial or financial relationships that could be construed as a potential conflict of interest.

## Abbreviations

AA(s): Amino acid(s)
APR(s): Acute-phase reactant(s)
BDNF: Brain-derived neurotrophic factor
BP(s): Binding – protein(s)
C/EBP: Enhancer-binding protein working as transcription factor
CSF: Cerebrospinal fluid
EGF: Epidermal growth factor
IGF: insulin-like growth factor
JAK/STAT: Signal transducer and activator of nuclear transcription
LBM: lean body mass
mRNA: messenger RNA
NGF: Nerve-growth factor
RARE: Retinoid acid response element
RCC: Retinol circulating complex
RQ: Respiratory quotient
STRA6: Protein coding gene stimulated by retinoic acid 6 involved in transport/homeostasis of retinol
TBG: Thyroxine-binding globulin.
